# Editorial: Bioactive agents for functionalization of biomaterials for precise tissue engineering

**DOI:** 10.3389/fbioe.2023.1141827

**Published:** 2023-01-31

**Authors:** Gang Wu

**Affiliations:** Department of Oral Cell Biology, Academic Centre for Dentistry Amsterdam (ACTA), University of Amsterdam (UvA) and Vrije Universiteit Amsterdam (VU), Amsterdam, Netherlands

**Keywords:** bioactive agents, precise tissue engineering, bone regeneration, skin disease, antibacterial, biofunctionalisation

Tissue engineering is a multidisciplinary technology to improve or replace biological tissues. Biomaterial scaffolds are an indispensable element in tissue engineering. A large variety of biomaterial scaffolds are widely adopted to facilitate the precise engineering of various tissues. The biomaterial scaffolds can be naturally derived or synthesized organic, inorganic, metallic, and hybrid materials. To facilitate precise tissue engineering, biomaterials can be further bio-functionalized by various bioactive agents. Bioactive agents can be naturally derived or synthetic cytokines, growth factors, extracellular vesicles, small-molecules, and peptides with one or multiple functions, such as osteoinduction, osteoconduction, anti-inflammation, anti-cancer, and anti-osteoclast. The methods to integrate bioactive agents into implants may include internal incorporation, electrostatic deposition, surface modification and entrapment.

To provide a scientific forum for the researchers in this field, we started this Research Topic—Bioactive agents for functionalization of biomaterials for precise tissue engineering in August 2021. Until now, 1 mini review, 6 reviews and 17 original research articles have been published with 191 authors involved. We hereby sincerely thank the excellent contributions of all these researchers to this successful Research Topic.

Similar as in our previous Research Topic (Advanced Biomaterials and Systems Releasing Bioactive Agents for Precise Tissue Regeneration), bone regeneration is still the hottest topic with 13 articles (1 mini review, 3 review and 9 original researches) published. One mini-review and one review summarized the key advances of two major bone-repairing components in clinic: calcium phosphate and barrier membrane. To facilitate pro-osteogenic functionalization of different biomaterials, various types of bioactive agents have been introduced and investigated in this Research Topic. The most investigated type in this Research Topic is plant-derived bioactive agents, such as k-carrageenan, sulfated carboxymethyl cellulose and quercetin, as well as animal-derived dopamine and sericin. Chen et al. reported platelet-activating biominerals enhanced injectable hydrogels with superior bioactivity for bone regeneration. Furthermore, one review summarized the application of traditional chinese medicine compound in functionalizing biomaterials for bone regeneration. Two research articles applied stem cells and M1 macrophage-derived exosomes as bioactive elements to promote osteogenic differentiation. Apart from these bioactive agents, bone regeneration may also be achieved by applying bio-reactive electrogenesis materials with electrophysical activity. In additional to bone tissue, cartilage regeneration was also achieved using Kartogenin-loaded GelMA hydrogel. Wang et al. reviewed the advances in regenerative sports medicine research.

Apart from bone and cartilage regeneration, three research articles discussed novel biofunctionalized biomaterials to treat skin diseases. Dong et al. reported the application of recombinant human-like collagen and fibronectin in treating acute skin wounds. Shi et al. applied adaptive gelatin microspheres as a stem cell delivery system to activate skin tissue regeneration in diabetic wounds. Peng et al. applied a daphnetin-loaded, modified hyaluronic acid-based dissolving microneedle to improve the treatment of psoriasis.

In addition to reports in the development of novel regenerative materials, a series of studies have focused on antibacterial functionalization of biomaterials. One review summarized the developments of the approaches to functionalize polyetheretherketone with antibacterial properties. Another review focused on the advancement of gallium and gallium-based compounds as antimicrobial agents. Liu et al. applied a novel astragaloside IV-loaded photothermal 2D nanosheet to treat infected wounds with its antibacterial and angiogenic properties. And Yan et al. presented a study on enhancing spontaneous antibacterial activity of δ-MnO2 by alkali Metals doping.

Apart from the above-mentioned Research Topic, original research articles have been presented to develop novel functionalized biomaterials for the treatment of pulmonary fibrosis, inner ear, and cancer. For example, Fang et al., developed a antifibrosis drug pirfenidone-functionalized mesoporous polydopamine for the treatment of pulmonary fibrosis. Interestingly, the authors also introduced an inhibitor of fibroblast activation protein, a 97 kDa type II transmembrane protein only overexpressed on the membrane of aberrantly activated fibroblast, onto the surface of polydopamine to facilitate the targeting to the lesion. Similarly, Hu et al. combined PLA polymer, malignant tumor-targeting T7 peptide, aggregation-induced emission-based fluorophore (tetrastyrene) and anti-tumor drug (Temozolomide) to develop a novel nanoprobe to target, bioimage and treat glioma. Luo et al. adopted β-cyclodextrin and oligoarginine peptide (Arg8)-modified dendrimer-entrapped gold nanoparticle as a drug delivery system to inner ear. All these studies showed a multi-functionalization pattern.

In summary, this Research Topic covers many recent advances in bioactive agents-functionalized biomaterials to treat various diseases, which contribute significantly to the progress of precise tissue regeneration.

